# *In Vitro* and *In Vivo* Anti-Angiogenic Activities of Panduratin A

**DOI:** 10.1371/journal.pone.0038103

**Published:** 2012-05-30

**Authors:** Siew-Li Lai, Shiau-Chuen Cheah, Pooi-Fong Wong, Suzita Mohd Noor, Mohd Rais Mustafa

**Affiliations:** 1 Centre of Natural Products & Drug Discovery (CENAR), Department of Pharmacology, Faculty of Medicine, University of Malaya, Kuala Lumpur, Malaysia; 2 Department of Molecular Medicine, Faculty of Medicine, University of Malaya, Kuala Lumpur, Malaysia; Charité-University Medicine Berlin, Germany

## Abstract

**Background:**

Targeting angiogenesis has emerged as an attractive and promising strategy in anti-cancer therapeutic development. The present study investigates the anti-angiogenic potential of Panduratin A (PA), a natural chalcone isolated from *Boesenbergia rotunda* by using both *in vitro* and *in vivo* assays.

**Methodology/Principal Findings:**

PA exerted selective cytotoxicity on human umbilical vein endothelial cells (HUVECs) with IC_50_ value of 6.91±0.85 µM when compared to human normal fibroblast and normal liver epithelial cells. Assessment of the growth kinetics by cell impedance-based Real-Time Cell Analyzer showed that PA induced both cytotoxic and cytostatic effects on HUVECs, depending on the concentration used. Results also showed that PA suppressed VEGF-induced survival and proliferation of HUVECs. Furthermore, endothelial cell migration, invasion, and morphogenesis or tube formation demonstrated significant time- and dose-dependent inhibition by PA. PA also suppressed matrix metalloproteinase-2 (MMP-2) secretion and attenuated its activation to intermediate and active MMP-2. In addition, PA suppressed F-actin stress fiber formation to prevent migration of the endothelial cells. More importantly, anti-angiogenic potential of PA was also evidenced in two *in vivo* models. PA inhibited neo-vessels formation in murine Matrigel plugs, and angiogenesis in zebrafish embryos.

**Conclusions/Significance:**

Taken together, our study demonstrated the distinctive anti-angiogenic properties of PA, both *in vitro* and *in vivo*. This report thus reveals another biological activity of PA in addition to its reported anti-inflammatory and anti-cancer activities, suggestive of PA’s potential for development as an anti-angiogenic agent for cancer therapy.

## Introduction

Angiogenesis, the formation of blood vessels from pre-existing vasculature, is a complex process that involves a cascade of events that are finely regulated under physiological conditions. Sustained, uncontrolled angiogenesis is associated with various pathological conditions including cancer, diabetic retinopathy, and rheumatoid arthritis [1]. Acquisition of the angiogenic phenotype is a rate-limiting step in tumor progression, wherein the tumor remains in a dormant state until it is able to stimulate blood vessel growth from nearby pre-existing capillaries [1], [2] in order to facilitate cancer cell progression and metastasis. Faced with problems associated with conventional anti-cancer drugs such as serious side effects, chemo- and radio-resistance, disease relapse, and metastases, the scientific community has looked to the development of alternative chemotherapeutic regimens, such as anti-angiogenic drugs. This anti-angiogenic strategy has been an important consideration for the development of cancer chemotherapeutics for the past three decades.

To date, validation of more than 40 anti-angiogenic agents in clinical settings is underway and several classes of anti-angiogenic agents have been approved by the Food and Drug Administration as anti-cancer drugs [3]. Nevertheless, current anti-angiogenesis therapy is still inadequate in improving overall survival of cancer patients [4]. In addition, limitations such as low efficacy, and development of resistance were evidenced with long term administration of these therapeutic agents, possibly through compensatory mechanisms such as upregulation of other pro-angiogenic factors or dysregulation in multiple signaling pathways [3], [4]. These limitations warrant more intensive effort in discovering and developing anti-angiogenic agents, especially of small molecules that target different angiogenesis pathways [5].

At present, much attention has been focused on natural product-based therapeutics, especially phytochemicals, owing to numerous reports that revealed the interference of phytochemicals on cancer-related pathways [6], [7]. Panduratin A (PA), a natural chalcone from *Boesenbergia rotunda* has been reported to exhibit anti-oxidant, anti-inflammatory and anti-cancer properties [8], [9], [10], [11], [12], but its anti-angiogenic effect has not been reported to date. To unravel the potential of PA as an anti-angiogenic agent, the present study investigated the *in vitro* effects of PA on various functions of human umbilical vein endothelial cells (HUVECs) which are pivotal to angiogenesis progression, before applying PA to *in vivo* models.

## Materials and Methods

### Ethics Statement

All experimental procedures were approved by the University of Malaya Animal Care & Ethics Committee. (Ethics Number: FAR/27/01/2010/0112/LYS(R)).

### Extraction and Isolation

**Figure 1:**
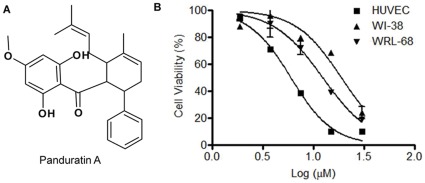
Selective cytotoxicity of PA on endothelial cells. (A) Chemical structure of Panduratin A (B) Dose-dependent cytotoxic effects of PA on HUVECs, WI-38, and WRL-68 cells as examined by MTT assay.

PA was isolated from *B. rotunda* (a voucher specimen with accession number KU0098 is deposited in the Phytochemistry Herbarium, University of Malaya, Kuala Lumpur) as previously described [8]. Briefly, methanolic crude extract was subjected to fractionation by preparative reversed-phase HPLC (Waters Nova-Pak C18 column, particle size 6 µm, 25×100 mm), using acetonitrile (0.1% formic acid) and water (0.1% formic acid) as mobile phases. The elution was performed with solvent gradient from 60% to 100% acetonitrile over 50 min at a flow rate of 12 mL/min. The purity of PA (Figure 1A) used in the present study was at >98% purity as determined by HRMS and nuclear magnetic resonance (1H-NMR) spectroscopy.

### Cell Culture

HUVECs were purchased from ScienCell (CA, USA), and the human hepatic epithelial cell line (WRL-68) and human fibroblast-like fetal lung cells (WI-38) were purchased from American Type Culture Collection (ATCC; VA, USA). HUVECs were cultured in Endothelial Cell Medium (ECM; ScienCell) supplemented with 5% heat-inactivated fetal bovine serum (FBS; ScienCell), 1% penicillin/streptomycin (ScienCell) and 1% Endothelial Cell Growth Supplement (ECGS; ScienCell). WI-38 and WRL-68 were maintained in Dulbecco’s Modified Eagle Medium (DMEM; Gibco, CA, USA) and Roswell Park Memorial Institute medium 1640 (RPMI; Gibco), respectively, supplemented with 10% heat inactivated FBS (Sigma-Aldrich, MO, USA) and 1% penicillin/streptomycin (Gibco). All cells were incubated at 37°C in humidified 5% CO_2_, 95% air.

### Cell Viability Assay

The effects of PA on the viability of HUVECs, WRL-68 and WI-38 were examined by MTT assay. Briefly, cells were seeded in a 96-well microtiter plate overnight, and allowed to reach ∼80% confluency after which indicated concentrations of PA were added. After 24 h, 50 µl of 3-(4,5-dimethylthiazol-2-yl)-2,5-diphenyl tetrazolium bromide (MTT; Sigma-Aldrich) solution (2 mg/ml) was added to each well and incubated for 2 h at 37°C. Then 100 µl of dimethyl sulfoxide (DMSO) was added to dissolve the MTT-formazan crystals formed by metabolically viable cells. The OD absorbance at 570 nm was detected and recorded using Plate Chameleon V microplate reader (Hidex, Turku, Finland).

### Real-Time Cell Growth Profiling

The growth kinetics of HUVECs, WRL-68 and WI-38 towards PA treatment were examined using the Real-Time Cellular Analyzer (RTCA) (xCELLigence system; Roche, Basel, Switzerland). This system utilizes non-invasive impedance readout to quantitate dynamic changes in cellular status such as growth in response to treatment. Cells were seeded at an empirically determined density in a 96-well gold–microelectrode array integrated E-plate (Roche) and incubated at 37°C in a humidified atmosphere of 5% CO_2._ The attachment and proliferation of cells were monitored every 5 min and the sensor impedance following cell attachment was expressed as an arbitrary unit called Cell Index (CI). Then, the cells were treated at the exponential phase and the kinetic responses of cells towards treatment were observed for 72 h in real-time mode. For Vascular Endothelial Growth Factor (VEGF)-induced cell proliferation, HUVECs were seeded and allowed to grow overnight prior to being serum-starved in starvation medium (1.5% FBS, ECGS free) for 6 h. Thereafter, indicated concentrations of PA and 50 ng/ml of VEGF (BD Biosciences, CA, USA) were co-administrated into the wells. The cell index was further monitored for 48 h. SU5416 (Sigma) was included as positive control.

### *In vitro* Capillary Tube Formation Assay

The effect of PA on morphogenesis of endothelial cells was investigated using capillary tube formation assay on Matrigel (BD Biosciences). Briefly, a cell density of 1.5×10^5^ cells/well was seeded on a Matrigel-pre-coated 96-well plate and treated with PA at concentrations of 3.5, 7, and 14 µM, respectively. VEGF at 1 ng/ml and Suramin (Sigma-Aldrich) at 40 µM were included as negative and positive controls, respectively. After 16 h, the medium was removed and the cells were fixed, permeabilized, and stained using DY554 phalloidin (Thermo Fisher Scientific, PA, USA) for F-actin and 4′-6-diamidino-2-phenylindole (DAPI; Thermo Fisher Scientific) for nuclei. Images of fluorescent labeled cells were acquired with the Cellomics Array Scan High Content Screening (HCS) Reader (Thermo Fisher Scientific) and analyzed using Tube Formation BioApplication algorithm (Thermo Fisher Scientific). This automated algorithm provides quantitative measurements of tube properties such as the number of connected tubes, tube area, and the Angiogenic Index.

### Scratch-Wound Directional Migration Assay

HUVECs were seeded at a cell density of ×10^5^ cells/well in a 96-well microtiter plate and allowed to grow into a confluent monolayer overnight. Then, the monolayer was scraped using a sterile 20–200 µl micropipette pipette tip to create a wound of ±1 mm width. The cells were washed twice with Hanks’ Balanced Salt Solution (HBSS; Sigma-Aldrich) and replaced with fresh medium containing indicated concentrations of PA. After 8 h, the cells were stained with Hoechst 33342 and Cellomics® whole cell stain green (Thermo Fisher Scientific). Cell migration was estimated by measuring the number of endothelial cells that had migrated from the edge of the wounded monolayer, as described elsewhere [13]. An area of 512×512 pixels of the wounded area was acquired using Cellomics Array Scan HCS Reader and the number of migrated cells was calculated by the HCS automated algorithm. Inhibition of migration was represented by a decrease in the number of cells in the image acquired relative to the untreated control. For each monolayer sample, three measurements were taken for three independent wounds.

### Chemoinvasion Assay

CIM-plate 16 (Roche) with Boyden-like chambers coupled with the RTCA xCELLigence system was used to examine the effects of PA on the chemotactic migration potential of HUVECs towards a chemoattractant. The assay was performed according to the manufacturer’s protocol, with minor modifications. HUVECs were serum starved for 4 h in basal ECM before being harvested and seeded onto the upper chamber of a CIM-plate. The PET membranes of CIM-plate 16 were pre-coated with 0.2 mg/ml of Matrigel. An uncoated control was included to measure the basal migration of HUVECs. Complete ECM, supplemented with FBS and ECGS, was placed in the lower chamber to act as a chemoattractant. In addition, another control with basal ECM in the lower chamber was included to monitor the random motility of the cells [14]. PA was added in both upper and lower chambers of the CIM-plate in order to provide ample time for the compound to diffuse into the cells and inhibit its target. The effects of PA on the chemoinvasion of HUVECs through Matrigel were monitored in real-time mode for 18 h.

### Cytoskeletal Rearrangement Study

The effects of PA on the actin and tubulin cytoskeletal systems of HUVECs were investigated by immunofluorescence. Briefly, HUVECs at ∼80% confluency were treated with PA for 16 h and stained with DY554-phalloidin for F-actin and anti-tubulin antibody for microtubules, respectively. Images were acquired on the Cellomics Array Scan HCS Reader and the effects on F-actin and microtubules were analyzed by Morphology BioApplication Algorithm (Thermo Fisher Scientific). Cytochalasin B (RBI; MA, USA), an F-actin depolymerizing agent and paclitaxel (Ascent Scientific; Bistrol, UK), a microtubule-stabilizing agent, were used as positive controls.

### Qualitative and Quantitative Measurement of Secretion of MMP-2

The effects of PA on MMP-2 secretion were examined quantitatively and qualitatively. HUVECs were seeded in complete medium and allowed to grow to ∼80% confluency. The cells were then washed thoroughly with HBSS to eliminate all medium residues, which was replaced with fresh serum free medium containing PA at 3.5 µM for the indicated periods before the collection of conditioned medium. The concentration of MMP-2 secreted by HUVECs in the conditioned media was measured using ELISA (Calbiochem, NJ, USA) according to manufacturer’s instructions. The conditioned media were also subjected to gelatin zymography (0.1% gelatin; 10% SDS-PAGE) under non-reducing conditions, as previously described [15], with slight modifications. After electrophoresis, the gels were washed twice for 30 min with renaturing buffer (2.5% Triton X-100) on a rotary shaker at room temperature. Then, the gels were incubated for 20 h at 37°C in developing buffer (50 mM Tris-HCl, 200 mM NaCl, 10 mM CaCl_2_, pH 7.8, 0.2% Brij 35). The gels were subsequently stained with staining solution (destaining solution with 0.1% Coomassie brilliant blue R-250) for 1 h and then destained in the destaining solution (45% methanol/10% acetic acid) until clear bands against a blue background were observed. The clear bands represented areas of gelatinolytic activities. Commercially available MMP standards (Calbiochem) and molecular marker (Invitrogen, CA, USA) were separated concurrently for MMP identification. Gel images were acquired on the Bio Rad Chemi XR Gel doc System (Bio-Rad, CA, USA).

### *In vivo* Matrigel Plug Assay

Matrigel plug assay in BALB/c mice was performed as described previously, with minor modifications [16], [17]. Female BALB/c mice (5–6 weeks old) were randomly divided into 4 different treatment groups and maintained under pathogen-free conditions. Mice were injected with 500 µl of Matrigel (BD Biosciences) containing heparin (64 U) and VEGF (150 ng/ml) with or without PA (5 µM). SU5416 (5 µM) was used as positive control. Another group with Matrigel plus **heparin** only was included as vehicle control. The mice were sacrificed after seven days and the Matrigel plugs were removed and photographed. To quantitate the formation of functional blood vessels, the amount of hemoglobin (Hb) was measured using the Drabkin hemoglobin assay as described previously [18].

### *In vivo* Zebrafish Assay

Zebrafish were maintained at 28°C on a 14/10 h day/night light cycle. Zebrafish embryos were generated by natural pair-wise mating. Fertilized embryos were maintained in embryo water (0.2 g/L ocean salt in distilled water) at 28.5°C. Healthy embryos at 24 hpf (21 somite stage) were manually dechorionated prior to being subjected to treatment by incubation in embryo water containing PA (15 µM). SU5416 (1 µM) was used as positive control. After treatment for 24 h, embryos were returned to normal embryo water for another 24 h. Then, embryonic blood circulation was videotaped using a camera mounted to a Zeiss inverted microscope after which the 72 hpf embryos were collected and fixed overnight at 4°C with 4% paraformaldehyde. Endothelial cells were visualized *in situ* by endogenous alkaline phosphatase staining [19].

### Statistical Analysis

Assays were performed in duplicate and three independent experiments were performed unless otherwise stated. Statistical significance were analyzed by using unpaired Student’s *t*-test or one-way analysis of variance (ANOVA) tests using Graphpad Prism v4.0 software (Graphpad Software, San Diego, CA, USA). Statistical significance is expressed as ***, *P*<0.001; **, *P*<0.01; *, *P*<0.05.

## Results

### Selective Cytotoxic and Cytostatic Effects of PA on HUVECs

**Figure 2:**
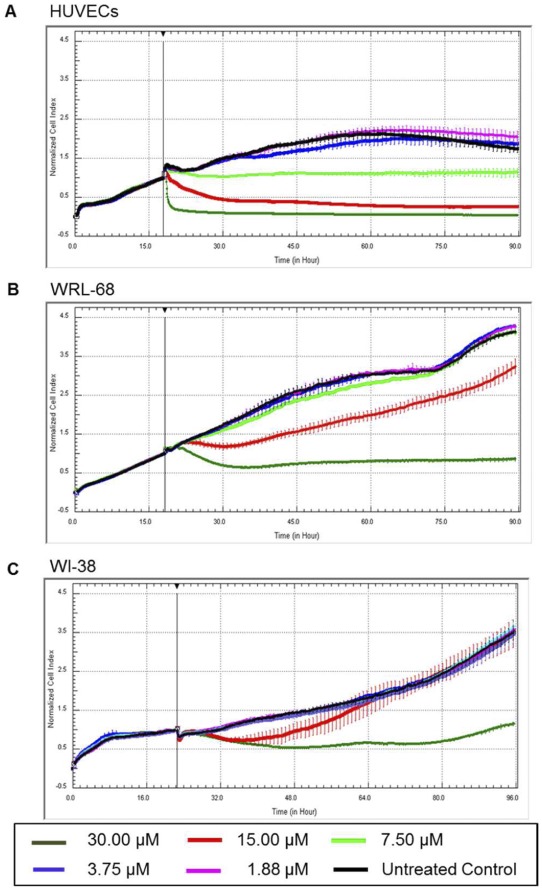
Selective cytotoxicity and anti-proliferative effects of PA on HUVECs. Dynamic growth and kinetic response of cells upon PA treatment were monitored using Real-Time Cell Analyzer. Representative Real-Time Cell Analysis Profiles of (A) HUVECs, (B) WRL-68, and (C) WI-38 cells treated with PA for 72 H.

Dose-dependent anti-endothelial effects of PA were investigated by both MTT assay (Figure 1B) and RTCA (Figure 2). In order to rule out the possibility of non-selective cytotoxic effects, PA was also tested in parallel on WI-38 human fibroblast cells and WRL-68 human hepatic epithelial cells. A summary of the IC_50_ values of HUVECs, WRL-68 and WI-38 cells at 24 h post-treatment with PA using MTT assay and RTCA is shown in Table 1. The IC_50_ values obtained were comparable between both methods. WI-38 and WRL-68 were found to be more resistant to the cytotoxic effect of PA, whereby their IC_50_ values increased by 4 and 3 folds, respectively, compared to HUVECs when determined by MTT assay.

**Table 1:** IC_50_ values of PA on HUVECs, WRL-68, and WI-38 derived from MTT assay and RTCA respectively.

	IC_50_ values (µM)	
	MTT	RTCA
	24 h	24 h
**HUVECs**	5.97±0.35	6.91±0.85
**WI-38**	18.86±0.22	15.17±0. 45
**WRL-68**	12.34±0.89	14.89±0.53

In contrast to the MTT assay that examines end point cytotoxicity, RTCA measures the dynamic growth and kinetic responses of cells following treatment with PA. PA at high dose (30 µM) exerted profound cytotoxicity effect on HUVECs, as indicated by a drastic decrease in CI values within 2 h post-treatment, wherein the growth of HUVECs failed to recover from the toxic insult (Figure 2A). On the contrary, a lower dose (7.5 µM) of PA exerted a cytostatic effect on HUVECs, as suggested by the unchanged CI values, which persisted over 72 h of treatment. PA at 1.88 and 3.75 µM, however showed no observable inhibitory effects on the growth of HUVECs and the cell proliferation was found to be parallel to that of the untreated control.

In addition, comparisons between the RTCA profiles of PA on HUVECs, WRL-68 and WI-38 clearly demonstrated the selective cytotoxicity of PA against endothelial cells. The profound cytotoxicity effect at 30 µM on HUVECs was not observed on WRL-68 and WI-38. Alternatively, PA at this concentration (30 µM) exerted cytostatic effects on WI-38 and WRL-68 cells with the growth of WI-38 subsequently recovering after 60 h of treatment. At 15 µM, WI-38 was found to have fully recovered from the initial cytotoxic effect, and began to re-proliferate, attaining growth similar to that of the untreated control after 42 h of treatment. WRL-68 also partially recovered from growth inhibition at 15 µM PA. These results showed that PA has selective toxicity against HUVECs and induces both cytostatic and cytotoxic effects as its concentration increased.

### PA Suppresses VEGF-induced Survival and Proliferation of HUVECs

**Figure 3:**
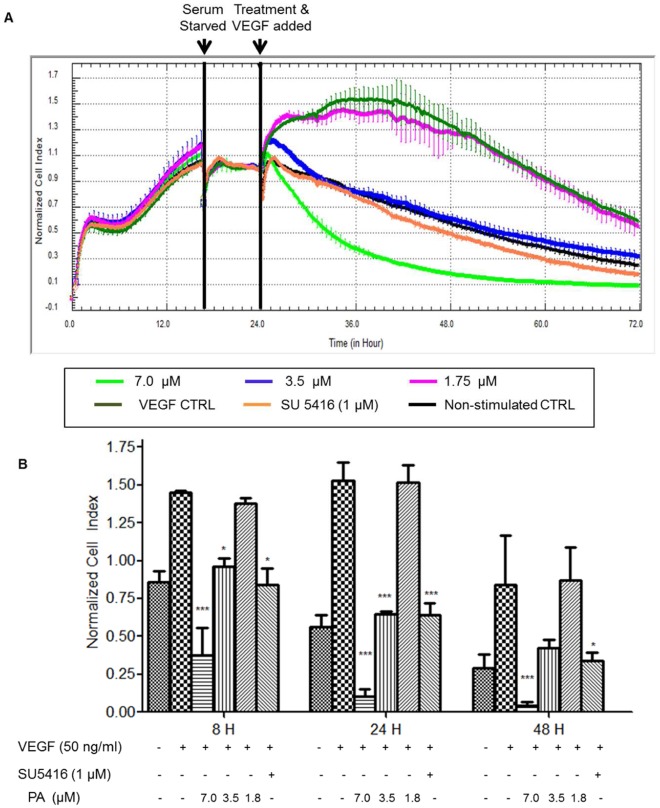
PA suppresses VEGF induced survival and proliferation on HUVECs. (A) Representative RTCA profile of VEGF induced growth and survival. HUVECs were seeded in an E-Plate and allowed to attach and grow overnight. At exponential growth phase, HUVECs were serum-starved for 6 h before stimulation with VEGF and co-treatment of PA (B) Normalized cell index of HUVECs at different time points upon co-administration with PA and VEGF. Data are expressed as means ± SEM of three independent experiments. Statistical significance is expressed as ***, *P*<0.001; **, *P*<0.01; *, *P*<0.05 versus VEGF control.

VEGF is an important mitogen and survival factor for endothelial cells. In response to angiogenic stimulation, endothelial cells enter into an active proliferative state. The effects of PA on VEGF-induced proliferation and survival of HUVECs were investigated. As shown in the RTCA profile (Figure 3A), non-stimulated HUVECs in starvation medium (1.5% FBS) ceased to proliferate and cell death ensued gradually, attributed to apoptosis induced by serum withdrawal, as previously reported [20]. Stimulation with VEGF (50 ng/ml) significantly promoted proliferation of quiescent HUVECs with maximal effect observed after 24 h, with a 2.7 fold increase in normalized cell index compared to non-stimulated HUVECs (Figure 3B). In addition, VEGF treatment also enhanced survival by rescuing HUVECs from cell death due to serum withdrawal.

Anti-endothelial effects of PA were found to be more potent against VEGF-induced HUVECs with IC_50_ values of 3.53 µM. Notably, treatment at 7 µM caused irreversible cytotoxicity in contrast to the cytostatic effect observed in the absence of VEGF stimulation (Figure 2A). Furthermore, co-administration of PA at 3.5 µM with VEGF effectively abrogated the protective effects of VEGF on endothelial cells apoptosis. This effect was similar to the activity of SU5416, a VEGFR2 (FLK-1/KDR) tyrosine kinase inhibitor [21] which was included as positive control. It is also noteworthy that PA did not further induce endothelial cell death at this dose, indicating PA selectivity in inhibiting VEGF-induced endothelial cell survival and proliferation, rather than generalized PA cytotoxicity. The data suggest that PA was able to suppress VEGF-induced survival and proliferation, and VEGF-induced HUVECs were more sensitive to the cytotoxic effects of PA.

### PA Inhibits Morphogenesis of HUVECs

**Figure 4:**
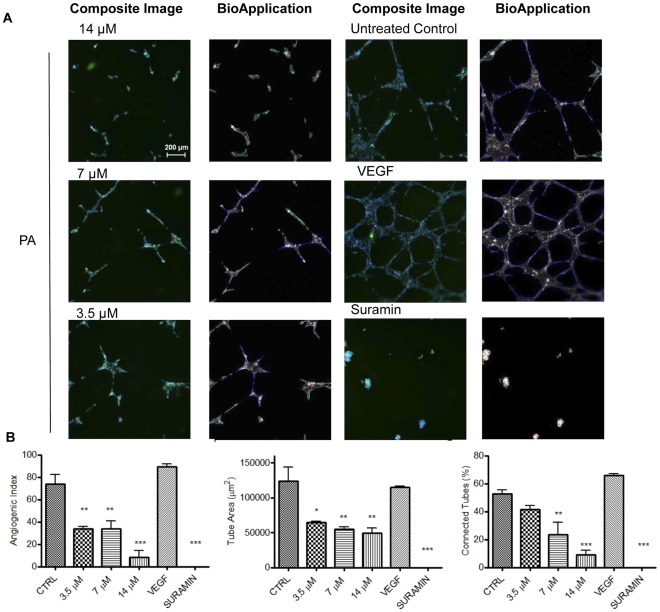
PA inhibits morphogenesis of endothelial cells. (A) Representative images of fluorescent-labeled HUVECs treated with different concentrations of PA. Greyscale fluorescence images are shown with colored overlays from the algorithm identifying the different entities measured: connected tubes are outlined in blue, unconnected objects are in aqua, nodes where the tube branches are marked by pink dots, and objects rejected from the quantitative analysis are in orange (B) Effects of PA on properties of tube formed (Angiogenic Index, Percentage of connected tubes and Tube area) as quantitated by Tube Formation BioApplication. Data are expressed as means ± SEM of three independent experiments. Statistical significance is expressed as ***, *P*<0.001; **, *P*<0.01; *, *P*<0.05 versus untreated control. Scale bar indicates 200 µm.

The effects of PA on endothelial cells tube formation were examined using an *in vitro* Matrigel HUVECs tube formation model. As depicted in Fure 4A, treatment by PA caused massive disruption of the capillary tubes network in contrast to the interconnecting capillary tube network in the untreated control. Tubes formed in the PA-treated wells were rather incomplete with shorter tube length and fewer branch points. VEGF, an endogenous pro-angiogenic factor and Suramin, a well-known angiogenic inhibitor were included as internal negative and positive controls, respectively. VEGF stimulated complete and well-formed networking of capillary tubes while Suramin completely impeded tube formation. Angiogenic Index (AI) is the measure of the percentage of the image area covered by tubes multiplied by 10 [22]. PA at 3.5, 7 and 14 µM significantly reduced the AI compared to untreated control. In addition, tube area and percentage of connected tubes as quantitated using an automated algorithm were also found to be negatively interrupted by PA treatment (Figure 4B). These results demonstrate that PA effectively inhibits HUVECs tube formation on Matrigel in a dose-dependent manner.

### Effect of PA on HUVECs Chemoinvasion

**Figure 5:**
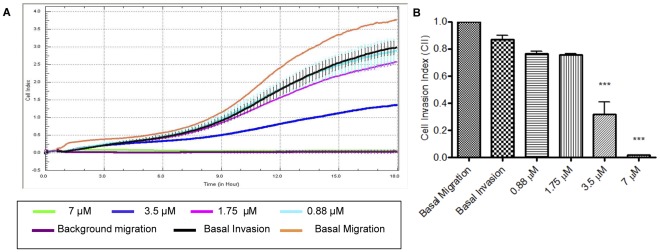
Effects of PA on Chemoinvasion as examined with Boyden-like CIM-plate **coupled with RTCA xCELLigence system.** (A) Representative figure of Real-time profile of HUVECs chemoinvasion (B) Cell invasion index (CII) of HUVECs at 18 h post-treatment with PA. Data are expressed as means ± SEM of three independent experiments. Statistical significance is expressed as ***, *P*<0.001 versus basal invasion.

During angiogenesis, endothelial cells degrade the basement membrane, invading and migrating towards an increasing gradient of chemoattractant [23]. Further investigations were done on the effects of PA on endothelial cell chemotaxis and invasion towards complete ECM as the chemoattractant. PET membranes of a CIM plate were pre-coated with Matrigel to create a barrier through which HUVECs must invade. Uncoated wells were also included to measure the basal migration (with complete ECM at the bottom chamber) and background migration (with serum free ECM at the bottom chamber). This would ensure the migration measured was chemotactic rather than random cell motility. The number of migrated cells was monitored in real-time mode using RTCA and expressed as Cell Invasion Index (CII), defined as the ratio of Cell Index of basal invasion to basal migration at a given time point. Basal invasion was defined as the invasion of untreated HUVECs towards complete ECM. Figure 5A shows the kinetic profile of HUVECs chemoinvasion towards complete ECM for 18 h, and the cell invasion index is illustrated in Figure 5B. PA inhibited the chemotactic invasiveness of HUVECs with IC_50_ of 2.61 µM. At 3.5 µM, the inhibitory effects of PA on HUVECs chemoinvasion were observed at ∼5 h post-treatment. However, exposure to PA at 7 µM completely impaired the migratory and invasive capability of HUVECs immediately after treatment commenced at the beginning of the assay. The data suggest that PA inhibited the chemoinvasion of PA in both time and dose-dependent manners.

### PA Inhibits the Migratory Ability of HUVECs

**Figure 6:**
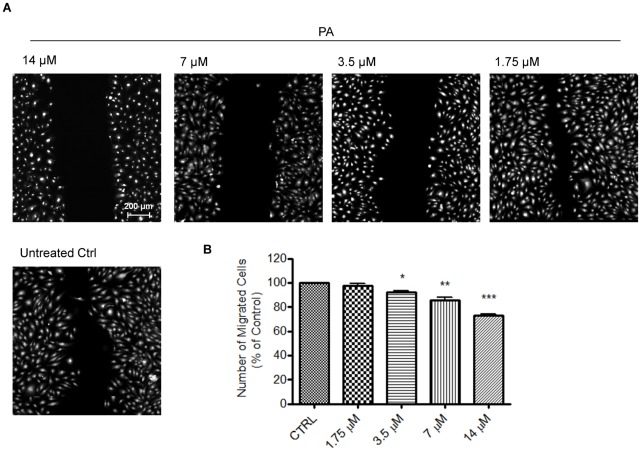
Effects of PA on HUVECs migratory ability as determined by scratch-wound assay. (A) Confluent monolayer of HUVECs was wounded and treated with either PA (1.75, 3.5, 7, 14) or medium alone (untreated control) for 8 h. The cells were then fixed and stained with Hoechst 33342 and Cellomics® whole cell stain green (B) Quantification of the number of migrated cells after 8 h exposure to indicated concentrations of PA. For each monolayer sample, three measurements were taken in three independent wounds. Percentage of inhibition was expressed using untreated wells at 100%. Data are expressed as means ± SEM of three independent experiments. Statistical significance is expressed as ***, *P*<0.001; **, *P*<0.01; *, *P*<0.05 versus untreated control. Scale bar indicates 200 µm.

Scratch-wound assay, a commonly used model [24], was performed to assess the effects of PA on the migratory ability of HUVECs. The effects on HUVECs migration were observed for 8 h, prior to the doubling time of HUVECs (∼24 h) in order to exclude any possible interference by HUVECs proliferation. In response to the wound, endothelial cells migrated into the denuded area, in a manner that mimicked the pattern of endothelial cell migration *in vivo* [25], [26]. As shown in Figure 6, untreated HUVECs migrated to the denuded area, but treatment with PA inhibited HUVECs migration in a dose-dependent manner, with PA doses of 3.5, 7, and 14 µM significantly reducing the percentage of migrated cells.

### PA Suppresses the Secretion and Activation of MMP-2

**Figure 7:**
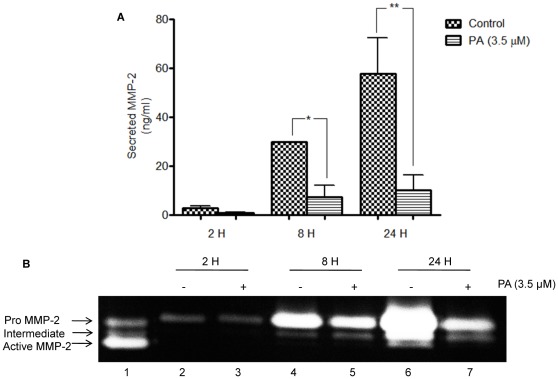
Effects of PA on HUVECs secretion of MMP-2. (A) Quantitative measurement of secreted MMP-2 by ELISA assay. Data are expressed as means ± SEM of two independent experiments. Statistical significance is expressed as *P*<0.01; *, *P*<0.05 versus untreated control (B) Qualitative analysis of expression of pro, intermediated and active MMP-2 using Gelatin zymography. Gel images were acquired on a Bio Rad Chemi XR Gel doc System.

MMP-2 is important in degrading the extracellular matrix during the course of endothelial cell invasion in angiogenesis. The correlation between the inhibitory effects of PA on HUVECs chemoinvasion through Matrigel, with the inhibition of MMP-2 secretion, was tested. Conditioned media of HUVECs exposed to PA (3.5 µM) at different time points were collected and quantitated using ELISA assay. As shown in Figure 7A, PA effectively suppressed the secretion of MMP-2, whereas untreated HUVECs continuously secreted MMP-2 into conditioned media, as depicted by the gradual increase of MMP-2 between 2 to 24 h. In contrast to the untreated control, PA treatment disrupted this pattern of time-dependent increase of MMP-2 secretion. This inhibitory PA effect on MMP-2 secretion was observed as early as 2 h post-exposure, where its expression was reduced by 3 folds. The suppression of MMP-2 persisted up to 24 h and resulted in a 6.5 fold reduction compared to the untreated control.

MMP-2 is secreted as inactive zymogen (pro MMP-2; 72 kDa) and is then activated to the intermediate form (64 kDa) and subsequently to the active form (active MMP-2; 62 kDa). Due to cross-reactivity in the ELISA assay, it was impossible to differentiate these different forms of MMP species. Gelatin zymography was used to further study the effects of PA on MMP-2 secretion and activation. The same conditioned media used for the ELISA assay was subjected to gelatin zymography and the result is illustrated in Figure 7B. Gelatin zymography separated MMP-2 into 3 bands; the pro MMP-2 (top), intermediate (middle) and active MMP-2 (bottom) (Lane 1, Figure 7B). It is observed that pro MMP-2 was the main species responsible for the increased MMP-2 secretion observed in the ELISA assay. A time-dependent increase in pro MMP-2 secretion was observed in untreated HUVECs (Lanes 2,4,6, Figure 7B). Treatment with PA for 24 h resulted in significant reduction of pro MMP-2 (Lane 7, Figure 7B) compared to untreated cells (Lane 6, Figure 7B). This result showed that PA had decreased the expression of pro MMP-2 in HUVECs which contributed, at least in part, to the invasive capability of HUVECs.

In addition, expressions of the intermediate MMP-2 species were observed at 8 and 24 h, while active MMP-2 only appeared at 24 h, signifying the gradual activation of pro MMP-2 to active MMP-2 by HUVECs. PA treatment significantly reduced expression in this moiety of MMP-2, suggesting that PA may also attenuate the activation of MMP-2.

### Effects of PA on the Cytoskeletal Systems of HUVECs

**Figure 8:**
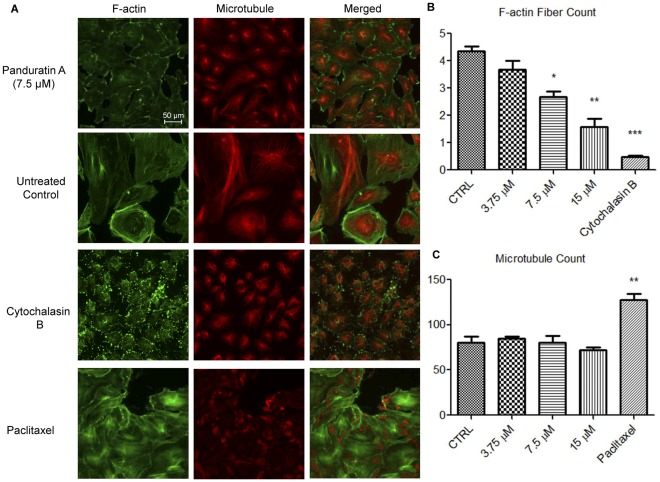
Effects of PA on cytoskeletals system of HUVECs. (A) HUVECs were treated with PA, paclitaxel, cytochalasin B, or medium alone (untreated control) for 16 h. HUVECs were fixed and stained with DY554-phalloidin for F-actin, and anti-tubulin antibody for microtubules, respectively. Images were acquired on Cellomics ArrayScan HCS Reader. Number of (B) F-actin (stress fiber), and (C) Microtubule fibers count were analyzed by Cellomics Morphology BioApplication. Data are expressed as means ± SEM of three independent experiments. Statistical significance is expressed as ***, *P*<0.001; **, *P*<0.01; *, *P*<0.05 versus untreated control. Scale bar indicates 50 µm.

Migration of HUVECs depends on the activation of several signaling pathways which eventually lead to cytoskeletal remodeling and ultimately, motility of cells, which prompted investigations into whether the observed anti-migration and anti-invasion effects of PA were due to disruption of actin and tubulin cytoskeletal systems. As shown in Figure 8A, paucity of stress fibers was observed in PA-treated HUVECs at 7.5 and 15 µM, in contrast to the well-developed, dense array of stress fibers in untreated controls. Analyses using Cellomics Morphology BioApplication algorithm showed 3 fold and 1.6 fold reduction in F-actin stress fiber count, respectively (Figure 8B), compared to untreated controls. Conversely, microtubule distribution and polymerization remained undisturbed in PA-treated HUVECs (Figure 8C). As a control, overnight treatment with cytochalasin B caused HUVECs actin depolymerization whereas paclitaxel, a microtubule-stabilizing agent [23], caused an increase in microtubule count. Collectively, these data suggest that the inhibition of HUVECs’ stress fiber formation may be one of the mechanisms exerted by PA in inhibiting HUVECs migration and invasion.

### PA Inhibits *in vivo* Angiogenesis in the Murine Matrigel Plug and Zebrafish Angiogenesis Models

**Figure 9:**
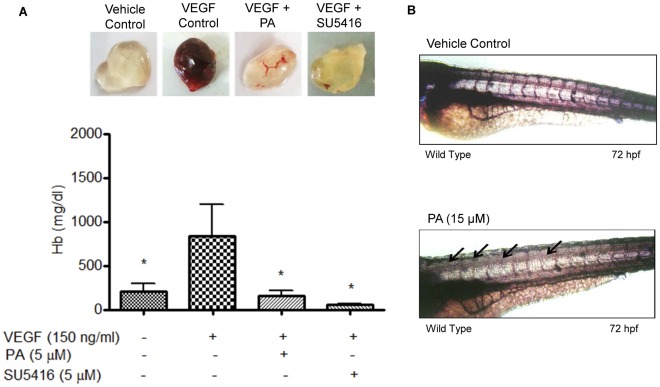
PA inhibits angiogenesis in *in vivo* murine Matrigel plug assay and *in vivo* zebrafish angiogenesis models. (A) PA inhibited angiogenesis induced by VEGF in the murine Matrigel plug model. Female BALB/c mice were injected subcutaneously with 500 µl of Matrigel containing VEGF (150 ng/ml) with or without PA (5 µM). VEGF plus SU5416 (5 µM) was used as positive control. The mice were sacrificed after seven days and the Matrigel plugs were removed and photographed. The degree of angiogenesis was determined indirectly by measuring the content of hemoglobin of the plugs using Drabkin’s assay. Data are expressed as means ± SEM. Statistical significance is expressed as P<0.05 versus VEGF induced control (n = 12–14) (B) PA exhibited anti-angiogenenic potential on the zebrafish angiogenesis model. Zebrafish embryos at 24 hpf were dechorionated and treated with PA (15 µM) or 0.1% DMSO for 24 h at 28.5°C. Thereafter, the treatment was removed and the embryos were maintained in normal embryo medium for another 24 h and fixed in 4% paraformaldehyde prior to *in situ* staining for endogenous alkaline phosphatase activity to visualize the blood vessels. Arrows denote inhibited ISVs. Embryos are of lateral view, with anterior to the left.

The anti-angiogenic potential of PA was subsequently validated in *in vivo* models. To determine whether PA could suppress or inhibit VEGF-induced angiogenesis in the Matrigel plug, mice were injected with 500 µl of Matrigel containing VEGF with or without PA, or Matrigel alone as negative control. In the presence of VEGF, the Matrigel plug appeared bright red, indicating that VEGF had induced and activated the mice endothelial cells to develop functional neo-vessels into the plug, whereas the Matrigel plug which lacked VEGF appeared pale. PA at 5 µM suppressed VEGF-induced neovascularisation, as observed from the reduction in neo-vesssel development into the plugs (Figure 9A). Hemoglobin content of the plugs were measured as an indirect indicator of angiogenesis. PA-containing plugs contained significantly lower hemoglobin content compared to VEGF-induced controls (Figure 9A). PA significantly inhibited or suppressed angiogenesis induced by VEGF in the murine Matrigel plug model.

Zebrafish embryos were exposed to PA at 24 hpf (21 somite stage), prior to the development of angiogenic vessels in order to determine the effects of PA on angiogenesis. The embryos were exposed to PA for 24 h, after which the embryos were returned to normal embryo medium for the next 24 h. The effects of PA on the intersegmental vessels (ISVs), the most easily observed angiogenic vessels, were monitored. As shown in the video (Video S1), the ISVs of control zebrafish embryo at 72 hpf were well developed, arranged in an extremely regular array, and achieved robust circulation [27]. Treatment with 7 µM PA did not exhibit anti-angiogenic effects (data not shown). The percentage of viable embryos after 24 h of treatment with 15 µM PA was 92.1% (Table 2). In the following 24 h of incubation in normal embryo medium, viability was further reduced to 74.6% (Table 2). In the positive control group, the percentage of viable embryos exposed to SU5416 was 95% (n = 20), with 100% of the viable embryos displaying disruption in blood flow (Table 3). However, after 24 h exposure to 15 µM PA (n = 63), disruption or loss of blood flow through ISVs Video S2() were observed in 75% of the embryos (Table 3), suggesting that PA inhibits or delays ISV development. In contrast, the percentage of viable embryos in the vehicle control group (n = 40) was 100% (Table 2), with all embryos displaying normal blood flow (Table 3). Endogenous alkaline phosphatase staining was done *in situ* to visualize the blood vessels. As shown in Figure 9B, ISVs of vehicle controls were well formed whereas embryos treated with PA showed incomplete ISV formation. This data suggests that PA inhibits neo-vascularization in zebrafish at the tested dose, in agreement with *in vitro* data.

**Table 2:** Percentage of embryo viability upon treatment with PA.

Treatment	Number of embryos (n)	Number of fatalities	Percentage of viability (%)
PA (15 µM)	63	5*; 11**	92.1%*; 74.6%**
SU5416 (1 µM)	20	1	95%
Vehicle control	50	0	100%

* Number of fatalities observed at 48 hpf (after 24 h treatment).

** Number of fatalities observed at 72 hpf (24 h treatment followed by 24 h recovery in normal embryo medium).

**Table 3:** Inhibition of angiogenesis in zebrafish embryo by PA.

Treatment	Number of embryos with inhibited vessels			Embryos with inhibited vessels (%)
	#Complete	##Partial	###Minor	
PA (15 µM)	1	32	14	75%
SU5416 (1 µM)	5	12	2	95%
Vehicle control	0	0	0	0%

# Complete inhibition of ISV blood flow.

## Inhibition of ≥4 ISV blood flow.

### Inhibition of ≤3 ISV blood flow.

## Discussion

Four decades ago, Folkman introduced the concept of targeting angiogenesis as a novel approach to the treatment of cancer [2]. Research in this area has since intensified to gain further understanding of the mechanisms and significance of angiogenesis in sustaining tumor growth and metastasis to facilitate the development of anti-angiogenic drugs. This includes the search for potential anti-angiogenic agents from natural resources. In this study, we demonstrated the anti-angiogenic potential of PA, a natural chalcone, by systematically characterizing its effects on various angiogenic functions of HUVECs, the most commonly used endothelial cell for *in vitro* angiogenesis assays. Angiogenesis progression depends on the increase in the viability and proliferation of endothelial cells [28], and we have shown that PA inhibits the growth of HUVECs in a dose-dependent manner. It is also noteworthy that PA is not cytotoxic at doses that inhibit angiogenic functions. Furthermore, fibroblast and epithelial cells were relatively resistant to PA at these doses, suggesting there is selectivity by PA against endothelial cells. VEGF is a major pro-angiogenenic factor that governs angiogenesis, in both physiological and pathological settings. Secretion of VEGF by tumor cells in response to hypoxia is an important factor that drives tumor angiogenesis and has been implicated in cancer progression [29]. We demonstrated that PA suppresses VEGF-induced survival and proliferation of HUVECs, implicating PA as an inhibitor of VEGF-driven angiogenesis. Capillary tube formation which involves attachment, matrix remodeling and morphogenesis, or differentiation of endothelial cells [30], is another prerequisite in angiogenesis progression. *In vitro*, endothelial cells plated on Matrigel will, once stimulated, attach, enter into growth arrest, and secrete proteases to invade the gel. The cells then migrate and differentiate into capillary tube networks in a manner that closely resembles the *in vivo* environment [30], [31]. Using a similar approach, we showed that PA disrupts *in vitro* tube formation, implicating PA with inhibitory effects on endothelial cell attachment, migration and invasion.

Migration of endothelial cells towards pro-angiogenic modulators is an integral feature of angiogenesis and this process can be simulated *in vitro* using Matrigel as an extracellular matrix barrier for invasion of HUVECs following the addition of a chemoattractant [14]. Migration can also be demonstrated using a scratch-wound assay, wherein *in vitro* denuded endothelial cells, when stimulated, will polarize towards the denuded space and migrate in a unidirectional fashion to make contact with migrating cells from the opposing wound edge [32], a mechanism that involves cell migration and cell-cell interactions [33]. In both models, it was shown that PA inhibits the migratory potentials of endothelial cells by preventing chemoinvasion of HUVECs towards serum-supplemented media, and migration of cells towards the denuded space in scratch wound assay. These data suggest that PA anti-migration activity is mediated through impedance of cell-cell interactions as well as a direct effect on endothelial cell locomotion apparatus, such as F-actin and microtubules.

Endothelial cell locomotion is a coordinated process which encompasses signal transduction, and cytoskeletal dynamics and re-organization. Stress fibers are bundles of actin filaments required for the contractions of the cell body during migration [34]. In sharp contrast to the dense array of stress fibers in untreated controls, stress fibers were scarce in PA-treated cells. PA had significantly interrupted the formation of actin stress fibers in endothelial cells, disrupting the contraction forces required for cell migration. Studies have unravelled the pivotal role of ROS in regulating endothelial cell migration and actin reorganization [35]. We propose that the inhibition of stress fiber formation in HUVECs observed is due to reported anti-oxidant properties of PA [36]. Sohn *et al.* [10] demonstrated the ability of reduction in intracellular ROS production by PA. The possible mechanism for the inhibitory effect by PA on migration is, at least in part, mediated through the reduction in endothelial intracellular ROS production, resulting in decreased stress fiber formation.

MMPs are a family of zinc-dependent endopeptidase that are capable of degrading components of the basement membrane and ECM, allowing endothelial cells to invade and migrate towards pro-angiogenic factors [37]. In intact cells, MMP-2 is secreted as an inactive zymogen (pro MMP-2; 72 kDA), which is further activated in extracellular milieu by the membrane-type MMP (MT1-MMP) with the aid of TIMP-2 to a 64 kDA active intermediate form [38], and a subsequent intermolecular autolytic cleavage leads to auto-activation to the 62 kDA activated MMP-2 [39]. Here, we showed that PA suppressed the secretion of MMP-2 and possibly attenuated its activation. The absence of active MMP-2 (62 kDA) moiety in PA-treated samples at 24 h could be due to physical interference by PA on the intermediate form of MMP-2, impeding its activation through an auto-proteolysis mechanism; or rather, this could be due to the decrease in intermediate forms, resulting in the activation of a minute amount of MMP-2 which could not be detected within current experimental conditions. MMP-2 has been strongly implicated in angiogenesis and is a critical factor for the switch to an angiogenic phenotype in tumors [40]. Intriguingly, endothelial-derived MMP-2 has been implicated in promoting cancer cell extravasation, thereby increasing the tumor’s metastatic potential [41]. We anticipate that the inhibitory effects demonstrated by PA against MMP-2 secretion will hold great pharmaceutical value as an anti-angiogenic agent for metastatic malignancies.

In addition, the anti-angiogenic potential of PA was also evidenced in two *in vivo* models. The use of zebrafish as an *in vivo* angiogenesis model has gained much attention due to its physiological similarities to mammals [42]. We were able to show that PA (15 µM) distinctly inhibited angiogenesis in zebrafish embryos following 24 h exposure. It was also observed that longer exposure to PA could result in toxicity to the embryos, while anti-angiogenic effects were not observed at a lower dose of PA (7 µM). These observations suggest that the anti-angiogenic effect on the embryos was partially due to an anti-endothelial effect at higher doses. It is also noted that the anti-angiogenic effect of PA was inferior to that of SU5416, which produced specific anti-angiogenic effects at 1 µM in the embryos. However, in the murine Matrigel plug model, treatment with a lower concentration of PA (5 µM) did result in inhibition of VEGF-induced blood vessel development into Matrigel plugs, alongside lower hemoglobin levels in the plugs. The differences observed between the two *in vivo* models could be attributed to different mechanisms of actions, and warrants further investigation to determine the exact anti-angiogenic mechanism of PA in different *in vivo* models.

In summary, our results strongly support the potential of PA as anti-angiogenic agent, with its multiple effects in inhibiting survival and proliferation of endothelial cells, morphogenesis, migration, chemoinvasion, stress fiber formation, and secretion and activation of MMP-2. To the best of our knowledge, these results represent first line evidence of the novel biological function of PA as an angiogenic inhibitor. Improvement of its current structure for more effective and specific derivatives should be considered for future development of PA as an anti-angiogenic agent.

## Supporting Information

Video S1 **Robust circulation of intersegmental vessels in control zebrafish embryos.** Embryos were maintained in normal embryos medium with 0.1% DMSO at 28.5°C. At 72 hpf, video of blood circulation was shot using a camera mounted to a Zeiss inverted microscope. Embryos are of lateral view, with anterior to the right.
(MPG)

Video S2 **PA disrupts the blood flow of intersegmental vessels in zebrafish embryo.** Embryos at 24 hpf were treated with PA (15 µM) for 24 h at 28.5°C. The treatment was then removed and embryos were maintained in normal embryo medium for another 24 h. At 72 hpf, video of blood circulation was shot using a camera mounted to a Zeiss inverted microscope. Embryos are of lateral view, with anterior to the right.
(MPG)

## References

[1] Munoz-Chapuli R, Quesada AR, Angel Medina M (2004). Angiogenesis and signal transduction in endothelial cells.. Cell Mol Life Sci.

[2] Folkman J (1971). Tumor angiogenesis: therapeutic implications.. N Engl J Med.

[3] Samant RS, Shevde LA (2011). Recent advances in anti-angiogenic therapy of cancer.. Oncotarget.

[4] Azam F, Mehta S, Harris AL (2010). Mechanisms of resistance to antiangiogenesis therapy.. Eur J Cancer.

[5] Jeong SJ, Koh W, Lee EO, Lee HJ, Bae H (2011). Antiangiogenic phytochemicals and medicinal herbs.. Phytother Res.

[6] Mojzis J, Varinska L, Mojzisova G, Kostova I, Mirossay L (2008). Antiangiogenic effects of flavonoids and chalcones.. Pharmacol Res.

[7] Ren W, Qiao Z, Wang H, Zhu L, Zhang L (2003). Flavonoids: promising anticancer agents.. Med Res Rev.

[8] Cheah SC, Appleton DR, Lee ST, Lam ML, Hadi AH (2011). Panduratin A inhibits the growth of A549 cells through induction of apoptosis and inhibition of NF-KappaB translocation.. Molecules.

[9] Rukayadi Y, Lee K, Han S, Yong D, Hwang JK (2009). *In vitro* activities of panduratin A against clinical Staphylococcus strains.. Antimicrob Agents Chemother.

[10] Sohn JH, Han KL, Lee SH, Hwang JK (2005). Protective effects of panduratin A against oxidative damage of tert-butylhydroperoxide in human HepG2 cells.. Biol Pharm Bull.

[11] Yun JM, Kweon MH, Kwon H, Hwang JK, Mukhtar H (2006). Induction of apoptosis and cell cycle arrest by a chalcone panduratin A isolated from *Kaempferia pandurata* in androgen-independent human prostate cancer cells PC3 and DU145.. Carcinogenesis.

[12] Yun JM, Kwon H, Hwang JK (2003). *In vitro* anti-inflammatory activity of panduratin A isolated from *Kaempferia pandurata* in RAW264.7 cells.. Planta Med.

[13] Pang X, Yi T, Yi Z, Cho SG, Qu W (2009). Morelloflavone, a biflavonoid, inhibits tumor angiogenesis by targeting rho GTPases and extracellular signal-regulated kinase signaling pathways.. Cancer Res.

[14] Albini A, Benelli R (2007). The chemoinvasion assay: a method to assess tumor and endothelial cell invasion and its modulation.. Nat Protoc.

[15] Toth M, Fridman R (2001). Assessment of Gelatinases (MMP-2 and MMP-9) by Gelatin Zymography.. Methods Mol Med.

[16] Kok TW, Yue PY, Mak NK, Fan TP, Liu L (2005). The anti-angiogenic effect of sinomenine.. Angiogenesis.

[17] Malinda KM (2003). *In vivo* matrigel migration and angiogenesis assays.. Methods Mol Med.

[18] Moore GL, Ledford ME, Merydith A (1981). A micromodification of the Drabkin hemoglobin assay for measuring plasma hemoglobin in the range of 5 to 2000 mg/dl.. Biochem Med.

[19] Habeck H, Odenthal J, Walderich B, Maischein H, Schulte-Merker S (2002). Analysis of a zebrafish VEGF receptor mutant reveals specific disruption of angiogenesis.. Curr Biol.

[20] Zoellner H, Hofler M, Beckmann R, Hufnagl P, Vanyek E (1996). Serum albumin is a specific inhibitor of apoptosis in human endothelial cells.. J Cell Sci.

[21] Fong TAT, Shawver LK, Sun L, Tang C, App H (1999). SU5416 is a potent and selective inhibitor of the vascular endothelial growth factor receptor (Flk-1/KDR) that inhibits tyrosine kinase catalysis, tumor vascularization, and growth of multiple tumor types.. Cancer Res.

[22] Ghosh RN, Lapets O, Haskins JR (2007). Characteristics and value of directed algorithms in high content screening.. Methods Mol Biol.

[23] Xiao H, Verdier-Pinard P, Fernandez-Fuentes N, Angeletti R, Fiser A (2006). Insights into the mechanism of microtubule stabilization by taxol.. Ejc Supplements.

[24] Lee JG, Kay EP (2006). FGF-2-induced wound healing in corneal endothelial cells requires Cdc42 activation and Rho inactivation through the phosphatidylinositol 3-kinase pathway.. Invest Ophthalmol Vis Sci.

[25] Rodriguez LG, Wu X, Guan JL (2005). Wound-healing assay.. Methods Mol Biol.

[26] Coomber BL, Gotlieb AI (1990). *In vitro* endothelial wound repair. Interaction of cell migration and proliferation.. Arteriosclerosis.

[27] Childs S, Chen JN, Garrity DM, Fishman MC (2002). Patterning of angiogenesis in the zebrafish embryo.. Development.

[28] Goodwin AM (2007). *In vitro* assays of angiogenesis for assessment of angiogenic and anti-angiogenic agents.. Microvasc Res.

[29] Kerbel RS (2008). Tumor angiogenesis.. N Engl J Med.

[30] Lawley TJ, Kubota Y (1989). Induction of morphologic differentiation of endothelial cells in culture.. J Invest Dermatol.

[31] Vucenik I, Passaniti A, Vitolo MI, Tantivejkul K, Eggleton P (2004). Anti-angiogenic activity of inositol hexaphosphate (IP6).. Carcinogenesis.

[32] kay Ja (2006). FGF-2-induced wound healing in corneal endothelial cells requires Cdc42 activation and Rho inactivation through the Phosphatidylinositol 3-Kinase Pathway..

[33] Coomber BL GA (1990). *In vitro* endothelial wound repair..

[34] Lamalice L, Le Boeuf F, Huot J (2007). Endothelial cell migration during angiogenesis.. Circ Res.

[35] Moldovan L, Mythreye K, Goldschmidt-Clermont PJ, Satterwhite LL (2006). Reactive oxygen species in vascular endothelial cell motility. Roles of NAD(P)H oxidase and Rac1.. Cardiovasc Res.

[36] Shindo K, Kato M, Kinoshita A, Kobayashi A, Koike Y (2006). Analysis of antioxidant activities contained in the *Boesenbergia pandurata Schult*. rhizome. Biosci. Biotechnol.. Biochem.

[37] Foda HD, Zucker S (2001). Matrix metalloproteinases in cancer invasion, metastasis and angiogenesis.. Drug Discov Today.

[38] Imai K, Ohuchi E, Aoki T, Nomura H, Fujii Y (1996). Membrane-type matrix metalloproteinase 1 is a gelatinolytic enzyme and is secreted in a complex with tissue inhibitor of metalloproteinases 2.. Cancer Res.

[39] Atkinson SJ, Crabbe T, Cowell S, Ward RV, Butler MJ (1995). Intermolecular autolytic cleavage can contribute to the activation of progelatinase A by cell membranes.. J Biol Chem.

[40] Fang JM, Shing Y, Wiederschain D, Yan L, Butterfield C (2000). Matrix metalloproteinase-2 is required for the switch to the angiogenic phenotype in a tumor model.. P Natl Acad Sci USA.

[41] Kargozaran H, Yuan SY, Breslin JW, Watson KD, Gaudreault N (2007). A role for endothelial-derived matrix metalloproteinase-2 in breast cancer cell transmigration across the endothelial-basement membrane barrier.. Clin Exp Metastasis.

[42] Kidd KR, Weinstein BM (2003). Fishing for novel angiogenic therapies.. Br J Pharmacol.

